# A recurrence‐predictive model based on eight genes and tumor mutational burden/microsatellite instability status in Stage II/III colorectal cancer

**DOI:** 10.1002/cam4.6720

**Published:** 2023-12-19

**Authors:** Zhaoya Gao, Zhiyi Wan, Pengfei Yu, Yan Shang, Guangsheng Zhu, Huiyuan Jiang, Yawei Chen, Shengzhou Wang, Fuming Lei, Wensheng Huang, Qingmin Zeng, Yanzhao Wang, Wanshui Rong, Yuming Hong, Qingkun Gao, Pengfei Niu, Zhichao Zhai, Ke An, Changmin Ding, Yunfan Wang, Guoli Gu, Xin Wang, Qingkai Meng, Shengwei Ye, Haiyi Liu, Jin Gu

**Affiliations:** ^1^ Department of General Surgery Peking University First Hospital Beijing China; ^2^ Genecast Biotechnology Co., Ltd. Wuxi City Jiangsu Province China; ^3^ Department of General Surgery Air Force Medical Center, Chinese People's Liberation Army Beijing China; ^4^ Department of Colorectal Surgery Cancer Hospital of China Medical University, Liaoning Cancer Hospital and Institute Shenyang Liaoning Province China; ^5^ Department of Gastrointestinal Surgery Hubei Cancer Hospital Wuhan Hubei Province China; ^6^ Department of Colorectal and Anal Surgery Shanxi Province Cancer Hospital/ Shanxi Hospital Affiliated to Cancer Hospital, Chinese Academy of Medical Sciences/Cancer Hospital Affiliated to Shanxi Medical University Taiyuan Shanxi Province China; ^7^ Department of Gastrointestinal Surgery Peking University Shougang Hospital Beijing China; ^8^ Department of Pathology Peking University Shougang Hospital Beijing China; ^9^ Key Laboratory of Carcinogenesis and Translational Research (Ministry of Education/Beijing), Department of Gastrointestinal Surgery Peking University Cancer Hospital & Institute Beijing China; ^10^ Peking‐Tsinghua Center for Life Sciences Peking University Beijing China; ^11^ Peking University International Cancer Institute Beijing China

**Keywords:** colorectal cancer, predictive model, recurrence risk, recurrence‐free survival, whole‐exome sequencing

## Abstract

**Background:**

Although adjuvant chemotherapy (ACT) is widely used to treat patients with Stage II/III colorectal cancer (CRC), administering ACT to specific patients remains a challenge. The decision to ACT requires an accurate assessment of recurrence risk and absolute treatment benefit. However, the traditional TNM staging system does not accurately assess a patient's individual risk of recurrence.

**Methods:**

To identify recurrence risk‐related genetic factors for Stage II/III CRC patients after radical surgery, we conducted an analysis of whole‐exome sequencing of 47 patients with Stage II/III CRC who underwent radical surgery at five institutions. Patients were grouped into non‐recurrence group (NR, *n* = 24, recurrence‐free survival [RFS] > 5 years) and recurrence group (R, *n* = 23, RFS <2 years). The TCGA‐COAD/READ cohort was employed as the validation dataset.

**Results:**

A recurrence‐predictive model (G8plus score) based on eight gene (*CUL9*, *PCDHA12*, *HECTD3*, *DCX*, *SMARCA2*, *FAM193A*, *AATK*, and *SORCS2*) mutations and tumor mutation burden/microsatellite instability (TMB/MSI) status was constructed, with 97.87% accuracy in our data and 100% negative predictive value in the TCGA‐COAD/READ cohort. For the TCGA‐COAD/READ cohort, the G8plus‐high group had better RFS (HR = 0.22, *p* = 0.024); the G8plus‐high tumors had significantly more infiltrated immune cell types, higher tertiary lymphoid structure signature scores, and higher immunological signature scores. The G8plus score was also a predict biomarker for immunotherapeutic in advanced CRC in the PUCH cohort.

**Conclusions:**

In conclusion, the G8plus score is a powerful biomarker for predicting the risk of recurrence in patients with stage II/III CRC. It can be used to stratify patients who benefit from ACT and immunotherapy.

## 1 BACKGROUND

Colorectal cancer (CRC) is a well‐known heterogeneous malignancy and is one of the most common tumors worldwide.^1^ Currently, the diagnosis and treatment of CRC is mainly driven by the evaluation of the TNM staging system.^2^ Although surgery and adjuvant chemotherapy (ACT) are widely used to treat patients with Stage II/III CRC, clinical decision‐making for ACT remains a challenge. The TNM staging approach cannot accurately evaluate the individual recurrence risk in patients. For instance, the patients with Stage IIB CRC have a poorer prognosis than those with Stage IIIA.^3^ Although ACT is generally advised for all patients with Stage III CRC, approximately 40%–70% of patients are cured by surgery alone.^4^, ^5^, ^6^ Only about 20% of patients with Stage III CRC benefit from ACT. Accurate screening of patients with a good prognosis might save them from the toxic side effects of ACT.^7^ Therefore, many molecular assays have been performed to discover potential biomarkers for the identification of patients at high risk to improve individualized management.^8^, ^9^, ^10^, ^11^, ^12^, ^13^, ^14^, ^15^, ^16^, ^17^, ^18^

These assays include gene mutation identification (such as microsatellite instability (MSI) and *POLE*),^8^, ^15^ gene expression (such as ColoPrint and Oncotype DX Colon),^9^, ^16^, ^17^ tumor immune microenvironment (Immunoscore),^13^, ^14^ and circulating tumor DNA (ctDNA).^10^, ^11^, ^12^, ^18^ Patients with MSI in stage II CRC have a favorable prognosis and do not benefit from ACT with 5‐fluorouracil.^15^ However, only about 15% of patients with Stage II tumors had MSI. Furthermore, MSI does not have a strong prognostic value for Stage III CRC.^5^, ^15^ Several gene expression signatures have been established to identify the recurrence risk of patients with CRC; however, their prognostic usefulness is limited.^19^ Importantly, gene expression profiling assays necessitate the use of formalin‐fixed and paraffin‐embedded (FFPE) tissue specimens. After surgery, ctDNAs have emerged as prognostic markers and have the potential to guide ACT decisions.^10^, ^11^, ^12^, ^18^ However, a major problem is the low negative predictive value (NPV) of ctDNA testing after surgery, even when the limit of detection is as low as 0.01%.^20^ Thus, identifying a powerful and robust biomarker to predict the prognosis of patients with Stage II/III CRC is of great clinical significance.

Here, we aimed to identify recurrence risk‐related genetic factors for patients with Stage II/III CRC using whole‐exome sequencing (WES) on FFPE specimens. A recurrence‐predictive model (G8plus score) based on eight genes (*CUL9*, *PCDHA12*, *HECTD3*, *DCX*, *SMARCA2*, *FAM193A*, *AATK*, and *SORCS2*) and TMB/MSI status was constructed and validated in the TCGA‐COAD/READ cohort. The G8plus score classifier was also used to examine the genetic and clinicopathological features of patients with CRC.

## 2 METHODS

### 2.1 Patients and data

We retrospectively investigated 60 patients with Stage II/III CRC who underwent radical surgery at five institutions (Peking University Shougang Hospital, Liaoning Cancer Hospital, Hubei Cancer Hospital, Shanxi Cancer Hospital, and Air Force Medical Center) between March 2010 and June 2019. After WES, 47 samples passed quality control and were used for subsequent analyses. Based on RFS, patients were grouped into a non‐recurrence group (NR, *n* = 24, RFS >5 years) and a recurrent group (R, *n* = 23, RFS <2 years). The clinicopathological features of all patients are shown in Data S1. In addition, 357 patients with Stage II/III CRC in the TCGA‐COAD/READ cohort were used to validate our prediction model. TCGA‐COAD/READ data were downloaded from TCGA Pan‐Cancer project (https://gdc.cancer.gov/about‐data/publications/pancanatlas). In the TCGA‐COAD/READ cohort, MSI‐H was identified as an MSIsensor score ≥3.5 or a MANTIS score >0.4.^21^, ^22^ CRC immunotherapy data were obtained from the Peking University Cancer Hospital (PUCH) cohort, which consisted of 25 patients with advanced CRC treated with immune checkpoint inhibitors.^23^ Durable clinical benefit (DCB) was defined as a complete response, partial response, or stable disease lasting ≥24 weeks.

### 2.2 DNA extraction

Tumor and paracancer DNA were extracted from FFPE specimens using a QIAamp DNA FFPE Tissue Kit (Qiagen, Hilden, Germany) following the manufacturer's instructions. The extracted DNA was quality checked using a Qubit 3.0 fluorimeter (Life Technologies, Eugene, USA).

### 2.3 WES

The DNA libraries were constructed and hybridized using a Twist Human Core Excome Kit (Twist Bioscience, South San Francisco, USA) following the manufacturer's protocol. Next, 150 bp paired‐end sequencing was performed on an Illumina NovaSeq 6000 sequencer (Illumina, Hayward, USA). Identification and annotation of somatic mutations were performed according to the methods described in previous reports.^24^, ^25^ Tumor mutation burden (TMB) was calculated as the count of non‐synonymous somatic mutations per million bases.^26^ The genetic variants in this cohort are shown in Data S2.

### 2.4 Calculation of the G8 and G8plus score

Genes with significantly different mutation frequencies between the R and NR groups were obtained using Fisher's exact test. The top 30 genes were selected based on *p*‐values. We assigned a score of 1 to each gene with mutations and a score of 0 to each gene without mutations. The final score for each patient was calculated by summing up the scores from all the genes in the patient. ROC analysis was performed to evaluate the predictive performance of these gene sets. Eight genes (*CUL9*, *PCDHA12*, *HECTD3*, *DCX*, *SMARCA2*, *FAM193A*, *AATK*, and *SORCS2*) were screened for use in the recurrence risk prediction model (G8 score) using iterative subtraction analysis. The G8‐high and G8‐low groups were stratified based on the appropriate cut‐off value in the ROC analysis. The G8plus score was calculated based on the G8 and TMB/MSI status. In this model, two points were subtracted for patients with TMB‐high and MSS. The cut‐off value was generated using ROC analysis.

### 2.5 Signature analysis

Mutational signature analysis was carried out by the mafTools package.^27^ Tertiary lymphoid structure (TLS) signature scores were calculated as the mean of the normalized values of 12 chemokine genes.^28^, ^29^ Infiltrating immune cell (IC) types were identified by single‐sample gene set enrichment analysis (ssGSEA).^30^, ^31^ Marker genes representing the 28 IC types were acquired from a previous report.^30^ We considered an IC type to be enriched in a patient group if the false discovery rate was ≤10%. The gene sets for the immunological signature were analyzed as described in previous studies.^30^, ^32^

### 2.6 Statistical analysis

Statistical analysis was performed using SPSS software version 22.0 (SPSS, Inc., Chicago, USA). Wilcoxon test was applied to compare non‐parametric datasets, including TMB and signature score. Fisher's exact test was applied to analyze the proportions between the two groups. Kaplan–Meier curves and the Cox method were used for survival analyses. A two‐sided approach was used for all statistical tests and the statistical significance value was set at *p* < 0.05.

## 3 RESULTS

### 3.1 Clinicopathologic and genetic characteristics

A total of 47 CRC patients undergoing surgery were successfully investigated using WES, including 23 patients who relapsed within 2 years (R group) and 24 patients who did not relapse within 5 years (NR group). The cancer types were colon cancer (43/47) and rectal cancer (4/47), and the stages were stage II (2/47) and III (45/47). The R and NR groups did not show differences in most clinical characteristics, including clinicopathological risk, MSI status, and ACT (Table 1). Survival analysis also showed that clinical risk based on T4 or N2 status was independent of recurrence‐free survival (RFS) (high‐risk, hazard ratio (HR) = 1.095, *p* = 0.79, Figure S1). Patients with poorly differentiated tumors had a significantly greater recurrence risk (Table 1). Patients having colon cancer and tumors on the right side also had a greater recurrence risk (Table 1).

**TABLE 1:** Clinical characteristics of patients (*n* = 47).

Clinicopathological features	NR group	R group	*p*‐Value
Gender			
Male	14	11	0.896
Female	10	12	
Age (years)			
≥65	7	7	1
<65	17	16	
Cancer type			
Colon Cancer	22	21	1
Rectal cancer	2	2	
Tumor location (Colon Cancer)			
Left	15	7	0.034
Right	7	14	
Differentiation			
Well/Moderate	21	13	0.024
Poor	3	10	
Nerve invasion			
No	20	16	0.441
Yes	4	7	
Stage			
II	0	2	0.234
III	24	21	
Margin			
Negative	24	23	1
Positive	0	0	
Adjuvant chemotherapy			
Yes	19	22	0.346
No	3	1	
NA	2	0	
Chemotherapy regimen			
Capecitabine	1	1	0.358
Capeox	8	14	
mFolfox6	10	7	
Duration of chemotherapy			
≤3 months	5	4	0.544
3–6 months	8	13	
≥6 months	6	5	
Smoking			
No	18	19	0.779
Yes	6	4	
Drinking			
No	21	19	0.951
Yes	3	4	
MSI			
MSI‐H	3	1	0.609
MSS	21	22	
T stage			
T2/3	12	18	0.069
T4	12	5	
N stage			
N0/1	18	13	0.227
N2	6	10	
Clinical risk (T4 or N2)			
High	17	12	0.310
Low	7	11	

*Note*: *p*‐Values were obtained using Fisher's exact test. R group, patients who relapsed within 2 years; NR group, patients who did not relapse within 5 years.

**FIGURE 1:**
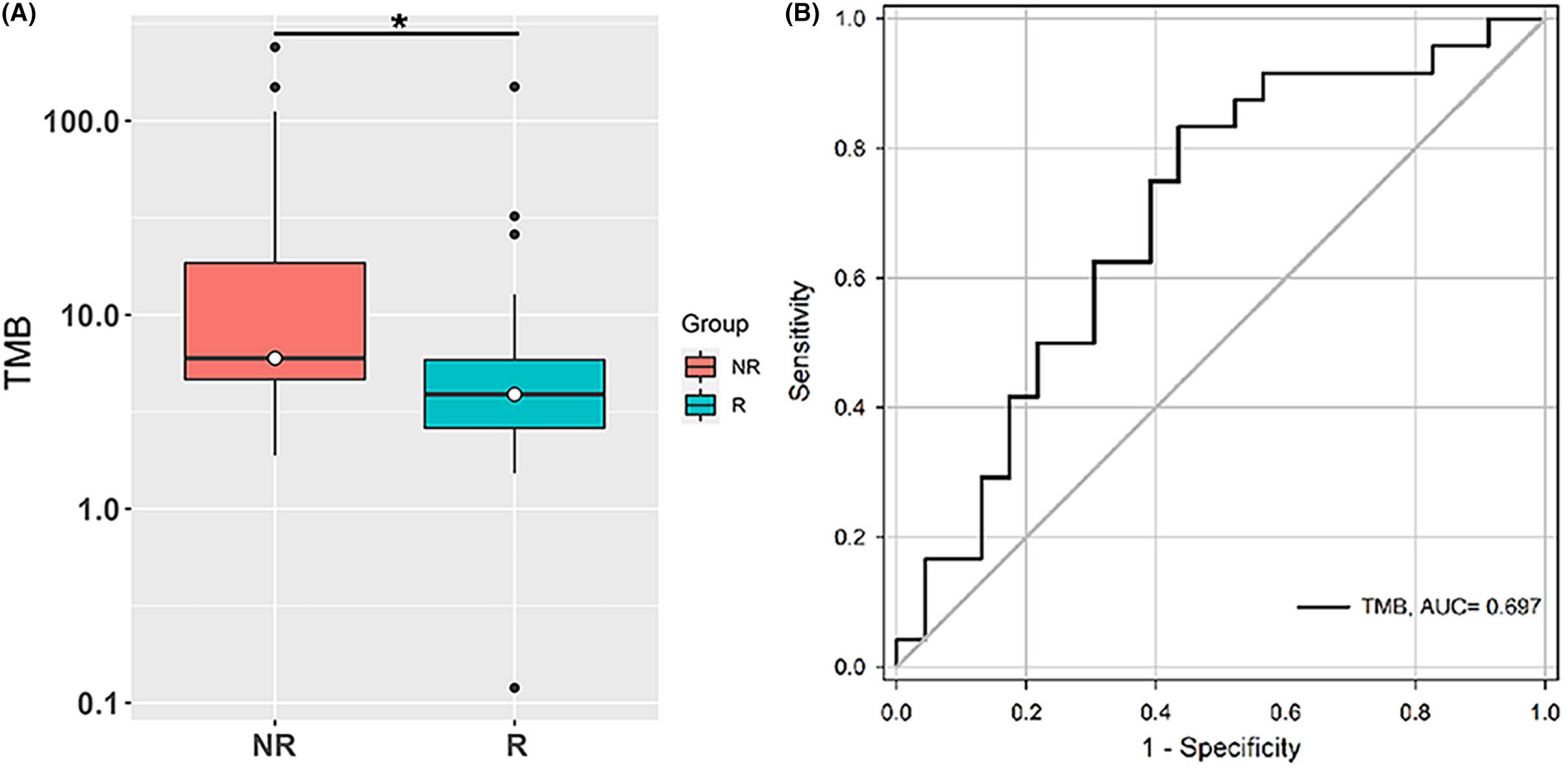
Tumor mutational burden (TMB) of CRC patients with or without recurrence. (A) Comparison of TMB between the R and the NR groups (Wilcoxon test). *p < 0.05. (B) Receiver operating characteristic (ROC) curve for prediction of recurrence by TMB in our data. AUC, area under curve. R, patients who relapsed within 2 years; NR, patients who did not relapse within 5 years.

Then, the genetic characteristics were compared between the R and NR groups (Figure S2). The top mutated genes, including *TTN*, *TP53*, *APC*, *MUC16*, and *KRAS*, were detected in both the R and NR groups (Figure S2a). However, the NR group had a significantly higher TMB than the R group (Figure 1A, *p* = 0.021). In addition, the NR group showed a significantly higher fraction of mutated samples in five signaling pathways than the R group (Figure S2b). Despite the significant difference in TMB between the two groups, receiver operating characteristic (ROC) analysis based on TMB indicated that the area under the curve (AUC) was only 0.697 (*p* = 0.020, Figure 1B).

### 3.2 Construction and validation of the recurrence‐predictive model (G8 score) based on 8 genes mutations

**FIGURE 2:**
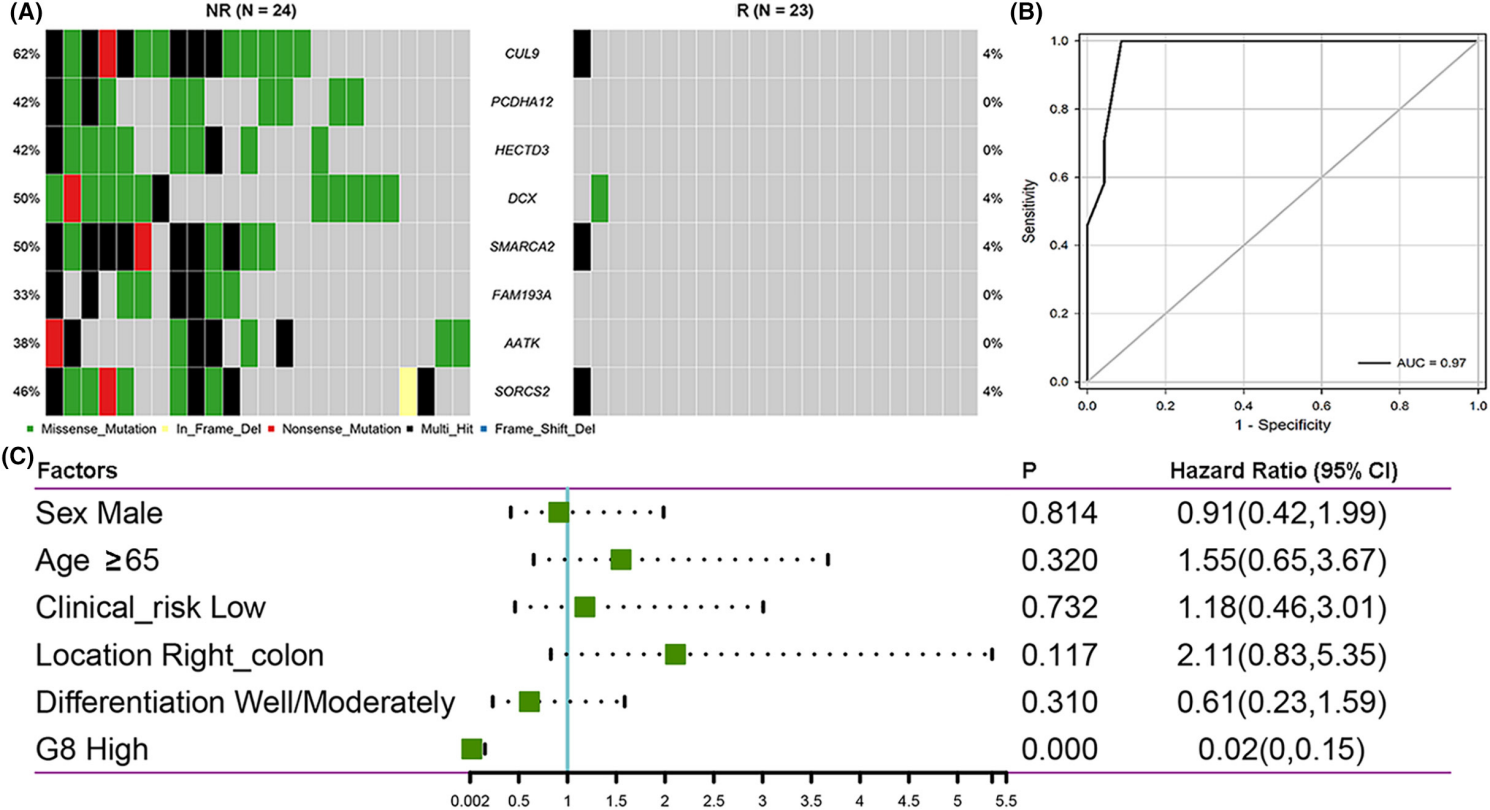
Construction of G8 score. (A) The distribution of the eight genes. (B) Receiver operating characteristic (ROC) curve for prediction of recurrence by G8 score in our data. AUC, area under curve. (C) Multivariate Cox analysis for RFS in our data. R, patients who relapsed within 2 years; NR, patients who did not relapse within 5 years.

A recurrence‐predictive model termed G8 score was further constructed based on the mutations in eight genes (*CUL9*, *PCDHA12*, *HECTD3*, *DCX*, *SMARCA2*, *FAM193A*, *AATK*, and *SORCS2*, Figure S3a). Mutations in these eight genes were detected almost exclusively in the NR group (Figure 2A). Moreover, these eight genes tended to co‐mutate (Figure S3b). The AUC score of the ROC based on the G8 score was 0.973, with a positive predictive value (PPV) of 100% and an NPV of 92.31% (*p* = 0.000, Figure 2B, Table S1). Multivariate Cox analysis (Figure 2C) showed that G8 score was an independent risk factor for RFS (HR = 0.020, *p* < 0.001).

The distribution of the eight genes in the TCGA‐COAD/READ cohort showed that G8‐high patients accounted for approximately 31% (64/204) of patients with stage II and 18% (28/153) of patients with Stage III (Figure S4a,b). Interestingly, the proportion of G8‐high patients decreased significantly from 31% in stage II to 18% in stage III and further significantly to 7% in stage IV (Figure S4b). In addition, G8‐high patients had higher MSI‐sensor scores and TMB (Figure S4a). Among Stage II/III CRC patients in the TCGA‐COAD/READ cohort, the proportion of MSI‐H in the G8 high and G8 low groups was approximately 48.9% (45/92) and 7.5% (20/265), respectively. There was also a co‐occurrence of these eight genes in the TCGA‐COAD/READ cohort (Table S2). However, they did not belong to the same pathway and had no direct connection with each other.

**FIGURE 3:**
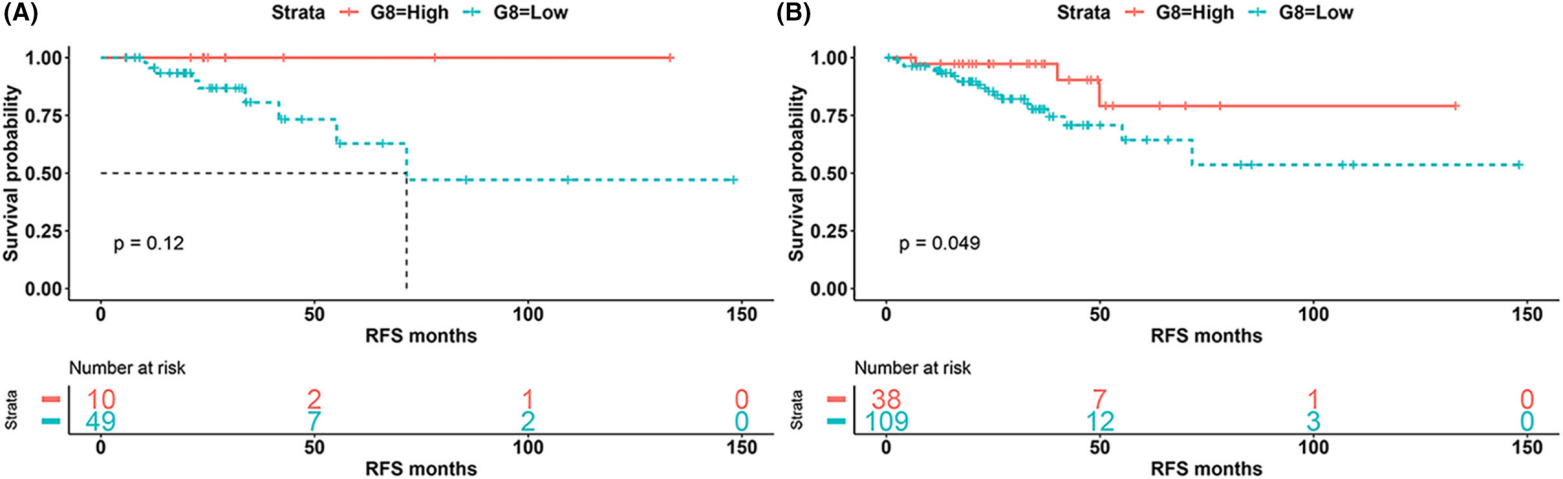
Validation of G8 score in the TCGA‐COAD/READ cohort. (A) Kaplan–Meier curve of recurrence‐free survival (RFS) for CRC patients with stage III based on G8 score in the TCGA‐COAD/READ cohort. (B) Kaplan–Meier curve of RFS for CRC patients with stage II/III based on G8 score in the TCGA‐COAD/READ cohort.

For patients with stage II/III CRC in the TCGA‐COAD/READ cohort, the G8 score accurately predicted patients who did not relapse within 2 years, with an NPV of 96.15% (Figure S4c). In the TCGA‐COAD/READ cohort, Kaplan–Meier curve analysis also indicated that the G8‐high group was associated with better RFS in patients with stage III CRC (HR <0.001, *p* = 0.120, Figure 3A), Stage IIB‐III (HR <0.001, *p* = 0.061, Figure S4d), and stage II/III (HR = 0.315, *p* = 0.049, Figure 3B). Notably, the prediction model based on the G8 score was not applicable to stage I patients in the TCGA‐COAD/READ cohort (HR = 0.095, *p* = 0.961, Figure S4e). Notably, in Stage II/III CRC patients with RFS information from the TCGA‐COAD/READ cohort, the proportions of MSI‐H and G8‐high were approximately 15.0% (22/147) and 25.9% (38/147), respectively. The G8 score was positively associated with MSI‐H, however, about 60.5% (23/38) of patients with G8 high scores in the validation cohort were still not MSI‐H.

### 3.3 Construction of the optimal predictive model (G8plus score) based on eight genes and TMB/MSI status

In our data (Data S1) and the TCGA‐COAD/READ cohort, the common feature of samples with negative prediction errors was TMB‐high and MSS. Mutational signature analysis showed that the most similar DNA signature in the negative‐prediction‐error samples was SBS10B (*POLE* exonuclease domain mutations), which was also unique to the negative‐prediction‐error samples in our data (Figure S5a). As expected, mutations in the *POLE* exonuclease domain were detected in all three samples, with negative prediction errors in our data and the TCGA‐COAD/READ cohort (Figure S5b).

**FIGURE 4:**
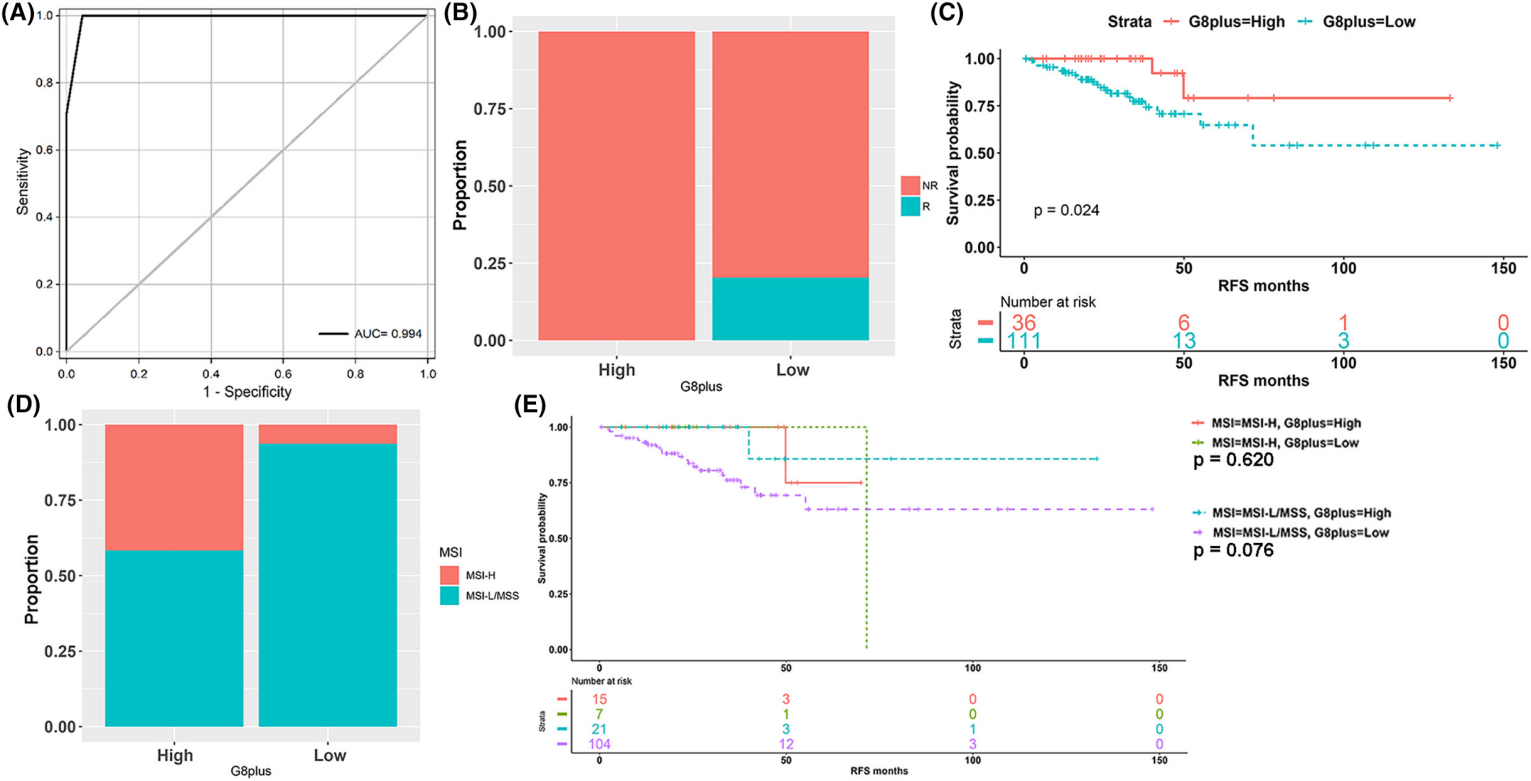
Construction and validation of G8plus score. (A) Receiver operating characteristic (ROC) curve for prediction of recurrence by G8plus score in our data. AUC, area under curve. (B) Predictive recurrence performance of the G8plus score in the TCGA‐COAD/READ cohort. (C) Kaplan–Meier curve of recurrence‐free survival (RFS) for CRC patients with stage II/III based on G8plus score in the TCGA‐COAD/READ cohort. (D) Comparison of MSI between G8plus high and G8plus low groups in the TCGA‐COAD/READ cohort. (E) Kaplan–Meier curve of RFS for CRC patients with Stage II/III based on G8plus score and MSI in the TCGA‐COAD/READ cohort.

Therefore, we further constructed an optimal predictive model (G8plus score) based on eight genes and the TMB/MSI status, with an accuracy of 97.87% in our data (Figure 4A) and an NPV (non‐recurrence within 2 years) of 100% in the TCGA‐COAD/READ cohort (Figure 4B). The proportions of patients with G8plus‐high score were approximately 24.5% (36/147). The G8plus‐high group was also associated with better RFS in Stage II/III CRC patients in the TCGA‐COAD/READ cohort (HR = 0.220, *p* = 0.024, Figure 4C). Among CRC patients with Stage II/III in the TCGA‐COAD/READ cohort, G8plus‐high patients had a greater proportion of MSI‐H (Figure 4D) and greater TMB value (Figure S6). It is important to note that MSI status was independent of RFS in stage II/III CRC patients in the TCGA‐COAD/READ cohort (Figure S7, HR = 0.450, *p* = 0.270). In Stage II/III CRC patients with MSI‐H, the G8plus score did not stratify patients for RFS (*p* = 0.620, Figure 4E). In Stage II/III CRC patients with MSS, the high G8plus score was also associated with better RFS (HR = 0.196), although the *p*‐value showed a near‐significant trend (*p* = 0.076, Figure 4E).

### 3.4 G8plus score as an indicator of immunotherapy in patients with CRC

**FIGURE 5:**
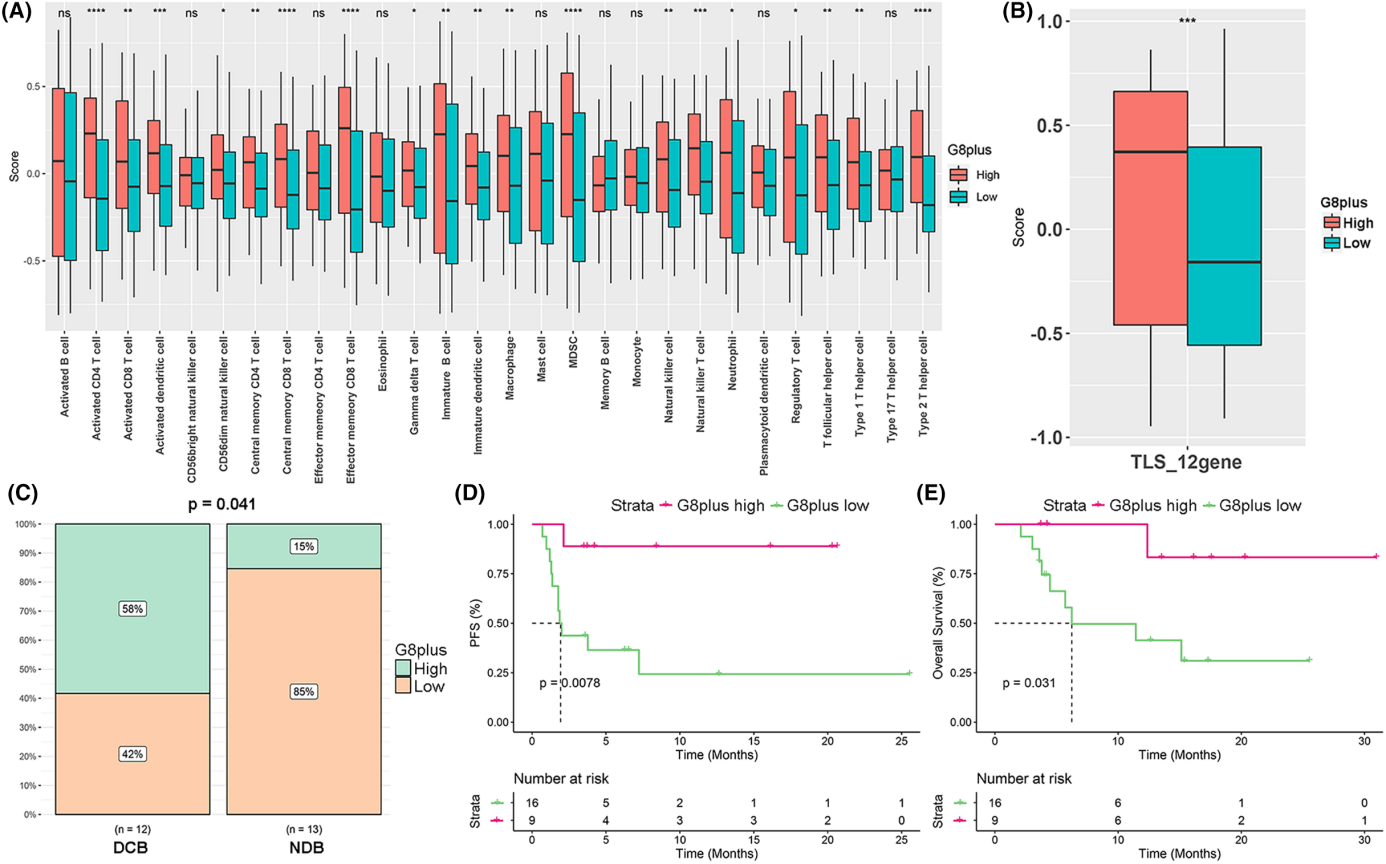
The G8plus score is a predict biomarker for immunotherapeutic in CRC patients. Comparison of infiltrated immune cell types (A) and tertiary lymphoid structure (TLS) signature score (B) between G8plus high and G8plus low groups in the TCGA‐COAD/READ cohort. (C) Comparison of the rate of durable clinical benefit (DCB) according to G8plus score in the PUCH cohort. NDB, no durable benefit. Kaplan–Meier curve of progression‐free survival (PFS, D) and overall survival (OS, E) for CRC patients with high and low G8plus score in the PUCH cohort.

We then explored the expression profile characteristics of G8plus‐high patients using RNA‐sequencing data from the TCGA‐COAD/READ cohort. SsGSEA showed that G8plus‐high tumors had significantly more infiltrated immune cell types, including activated CD8 T cells, activated CD4 T cells, activated dendritic cells, central memory CD8 T cells, effector memory CD8 T cells, and Type 1 T helper cells (Figure 5A). G8plus‐high tumors also had a significantly higher TLS signature score (Figure 5B), higher immune checkpoint gene expression (Figure S8a), and higher immunological signature scores (Figure S8b). These data indicated that the G8plus score may be an indicator of immunotherapy in patients with CRC. The G8‐high group was associated with better DCB (*p* = 0.041), PFS (HR = 0.100; *p* = 0.008), and OS (HR = 0.140; *p* = 0.031) in the PUCH cohort (Figure 5C–E).

## 4 DISCUSSION

Accurate prediction of recurrence risk is of vital importance in the clinical management of patients with Stage II/III CRC. However, current clinicopathological risk factors are imperfect, leading to misclassification and unnecessary postoperative interventions.^10^ Significant efforts have been performed to investigate the biomarkers that can predict recurrence risk. Moreover, it is currently not possible to predict recurrence from genomic variations in primary tumor tissues owing to the absence of validated biomarkers.^33^ In this study, we constructed a genomic classifier for recurrence risk for patients with Stage II/III CRC, called the G8plus score, consisting of eight genes and TMB/MSI status. In both our data and the TCGA‐COAD/READ cohort, the G8plus score was highly accurate in identifying patients who did not relapse within 2 years, with an NPV of 96% and 100%, respectively.

Although our data focused on Stage III colon cancer, the G8 score can be expanded to apply to all Stage II/III CRC in the TCGA‐COAD/READ cohort. Especially in patients with Stage IIB‐III CRC, the G8 score stratification had an extremely strong HR (HR <0.001), although the *p*‐value showed a moderate trend toward significance (*p* = 0.061). MSI is the most significant prognostic biomarker in patients with Stage II CRC and is widely used by the clinicians to select adjuvant therapy.^15^, ^34^ However, in the TCGA‐COAD/READ cohort, MSI status was not associated with RFS in patients with Stage III CRC. Although the G8plus score did not stratify Stage II/III CRC patients with MSI‐H, it does stratify Stage II/III CRC patients with MSS. The G8plus score was a stronger predictor of recurrence risk than MSI. In addition, G8‐high patients accounted for approximately 31% of the Stage II patients in the TCGA‐COAD/READ cohort, which was also higher than the proportion of patients with Stage II MSI‐H. Therefore, the G8 score can screen out more high‐risk patients than MSI. The proportion of G8‐high patients in the TCGA‐COAD/READ cohort decreased significantly from 31% in Stage II to 7% in Stage IV, implying a better prognosis for G8‐high patients.

Notably, the G8plus score seems to work in Stage II/III CRC patients with MSS but not in those with MSI‐H. However, the cases analyzed in this study are limited. More studies are needed to confirm this finding, especially in Stage III CRC patients. Unlike Stage II CRC, the prognostic role of MSI status in Stage III CRC remains controversial.^5^, ^15^ Our results showed that 68.18% (15/22) of patients with MSI‐H were G8plus high in Stage II TCGA‐COAD/READ cohort, while only 20% (1/5) of patients with MSI‐H were G8plus high in Stage III TCGA‐COAD/READ cohort. Therefore, the stratification effect of the G8plus score in Stage III CRC with MSI needs special attention.

In our data and the TCGA‐COAD/READ cohort, the common feature of the samples with negative prediction errors based on the G8 score was TMB‐high and MSS, which may be due to mutations in the *POLE* exonuclease domain. In the G8plus score model, patients with deductions based on TMB and MSI status were fully classified as the COAD/READ‐POLE subtype in the TCGA‐COAD/READ cohort. Mutations in the *POLE* exonuclease domain have been detected in approximately 1–3% of patients with CRC.^35^, ^36^ A previous study found that stage II CRC patients with *POLE* exonuclease domain mutations, regardless of MSI status, had markedly better RFS than those with *POLE* wild type in stage II CRC patients.^8^ However, our data shows that this argument remains controversial. Part of the reason for this difference may be the difference in the patient stage and MSI status. In stage II/III CRC patients with RFS information from the TCGA‐COAD/READ cohort, about 80% (4/5) of patients with *POLE* mutations were MSI‐H, whereas about 20% (1/5) of patients with *POLE* mutations were MSS. The RFS of patients with *POLE* mutations and MSS requires further attention.

G8plus‐high tumors had a higher proportion of MSI‐H, TMB, infiltrated immune cell types, higher immunological signature scores, and a higher TLS score, suggesting that the G8plus score may be a predictor of response to immunotherapy in patients with CRC, as confirmed in the PUCH cohort. CD8 T cell–mediated adaptive immunity is promoted by T helper type 1 (Th1) cells that produce cytokine interferon gamma.^37^ Th1 helper cells, cytotoxic T cells, macrophages, and associated cytokines can coordinate the elimination of tumor.^38^, ^39^ Th1 adaptive immunity has a beneficial effect on clinical outcomes in CRC.^40^ The trafficking properties and durable antitumor capacity of memory T cells have a core role in controlling cancer recurrence.^41^ Memory T cells maintain the ability to recognize colon cancer in the absence of antigen.^40^ Here, we showed a positive correlation between the G8plus score and Th1 cells, cytotoxic and memory T cells, macrophages, as well as a low recurrence risk. The higher proportion of infiltrating immune cells, especially memory T cells, may explain the low risk of recurrence in patients with high G8plus score. In addition, the immune microenvironment of patients with high G8plus score also suggested that the G8plus score could be used to stratify patients who might benefit from immunotherapy in CRC. Recently, neoadjuvant immunotherapy has been used to treat localized CRC patients with MSI‐H.^42^ In the future, the G8plus score may be useful in screening patients sensitive to neoadjuvant immunotherapy in Stage II/III CRC.

Several gene expression profile assays have been developed to identify prognostic biomarkers for patients with Stage II/III CRC.^9^, ^16^, ^17^ However, instability in the quality of RNA extracted from FFPE specimens limits the clinical application of these assays. The G8plus score, which is based on DNA testing of primary tumor tissue, can be easily applied to clinical testing. Several recent reports have shown that ctDNA is a powerful predictor of CRC recurrence.^10^, ^11^, ^12^, ^43^ However, the NPV for recurrence based on ctDNA is usually low, despite the high PPV. Our data showed that the G8plus score had a high NPV, suggesting that the G8plus score was well complemented by the ctDNA analysis. Therefore, the combination of the G8plus score and ctDNA testing may theoretically provide a more accurate assessment of the recurrence risk and thus stratify patients who may benefit from ACT. According to TCGA‐COAD/READ data, G8plus high patients account for just 18% of Stage III patients, limiting clinical use of the G8plus score. However, the recurrence‐free rate for patients with Stage III CRC has been reported to be about 40%–70%.^6^, ^44^

## 5 CONCLUSIONS

Taken together, we constructed a recurrence‐predictive model (G8plus score) based on DNA testing in primary tumor tissues and tested it using the TCGA‐COAD/READ cohort. The prognostic significance of classification by the G8plus score was superior to that of classical clinicopathological risk and MSI for RFS. Furthermore, the G8plus score is not only beneficial for predicting RFS but also for directing immunotherapy in advanced CRC patients.

## AUTHOR CONTRIBUTIONS

**Zhaoya Gao:** Data curation (equal); formal analysis (equal); project administration (equal); resources (equal); writing – original draft (equal). **Zhiyi Wan:** Data curation (equal); formal analysis (lead); visualization (lead); writing – original draft (equal). **Pengfei Yu:** Data curation (equal); investigation (equal); project administration (equal); resources (equal). **Yan Shang:** Data curation (equal); investigation (equal); project administration (equal); resources (equal). **Guangsheng Zhu:** Data curation (equal); investigation (equal); project administration (equal); resources (equal). **Huiyuan Jiang:** Data curation (equal); investigation (equal); project administration (equal); resources (equal). **Yawei Chen:** Formal analysis (equal); visualization (equal). **Shengzhou Wang:** Formal analysis (equal); visualization (equal). **Fuming Lei:** Project administration (equal); resources (equal). **Wensheng Huang:** Project administration (equal); resources (equal). **Qingmin Zeng:** Project administration (equal); resources (equal). **Yanzhao Wang:** Project administration (equal); resources (equal). **Wanshui Rong:** Project administration (equal); resources (equal). **Yuming Hong:** Project administration (equal); resources (equal). **Qingkun Gao:** Project administration (equal); resources (equal). **Pengfei Niu:** Project administration (equal); resources (equal). **Zhichao Zhai:** Project administration (equal); resources (equal). **Ke An:** Project administration (equal); resources (equal). **Changmin Ding:** Project administration (equal); resources (equal). **Yunfan Wang:** Project administration (equal); resources (equal). **Guoli Gu:** Conceptualization (supporting); resources (equal); supervision (equal); writing – review and editing (equal). **Xin Wang:** Conceptualization (supporting); resources (equal); supervision (equal); writing – review and editing (equal). **Qingkai Meng:** Conceptualization (supporting); resources (equal); supervision (equal); writing – review and editing (equal). **Shengwei Ye:** Conceptualization (supporting); resources (equal); supervision (equal); writing – review and editing (equal). **Haiyi Liu:** Conceptualization (supporting); resources (equal); supervision (equal); writing – review and editing (equal). **Jin Gu:** Conceptualization (lead); funding acquisition (lead); supervision (lead); writing – review and editing (lead).

## FUNDING INFORMATION

This work was supported by National Natural Science Foundation of China (82073223) and Capital's Funds for Health Improvement and Research of China (CFH 2020‐1‐6041).

## CONFLICT OF INTEREST STATEMENT

The authors declare no competing interests.

## ETHICS STATEMENT

The study was performed in accordance with the Declaration of Helsinki and approved by the Ethics Committee of Peking University Shougang Hospital. Written informed consent was provided by all participants.

## Supporting information


Data S1:

Data S2:

Data S3:

## Data Availability

All data analyzed in this study are included in this published article and its supplementary information document.
