# Supplementary material for: A recurrence‐predictive model based on eight genes and tumor mutational burden/microsatellite instability status in Stage II/III colorectal cancer
Source: Cancer Med. 2023 Dec 19;13(1):e6720. doi: 10.1002/cam4.6720 (PMC10807589; doi:10.1002/cam4.6720)
Supplement: Supplementary file 3 — Data S3: [file CAM4-13-e6720-s001.docx]

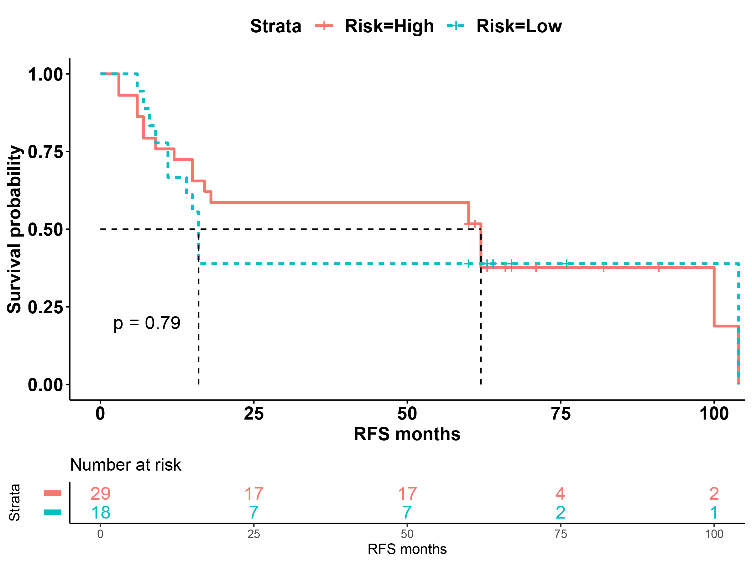


**Figure S1.** Kaplan–Meier curve of recurrence-free survival (RFS) based on clinical risk in our data. Clinical risk: T4 or N2.


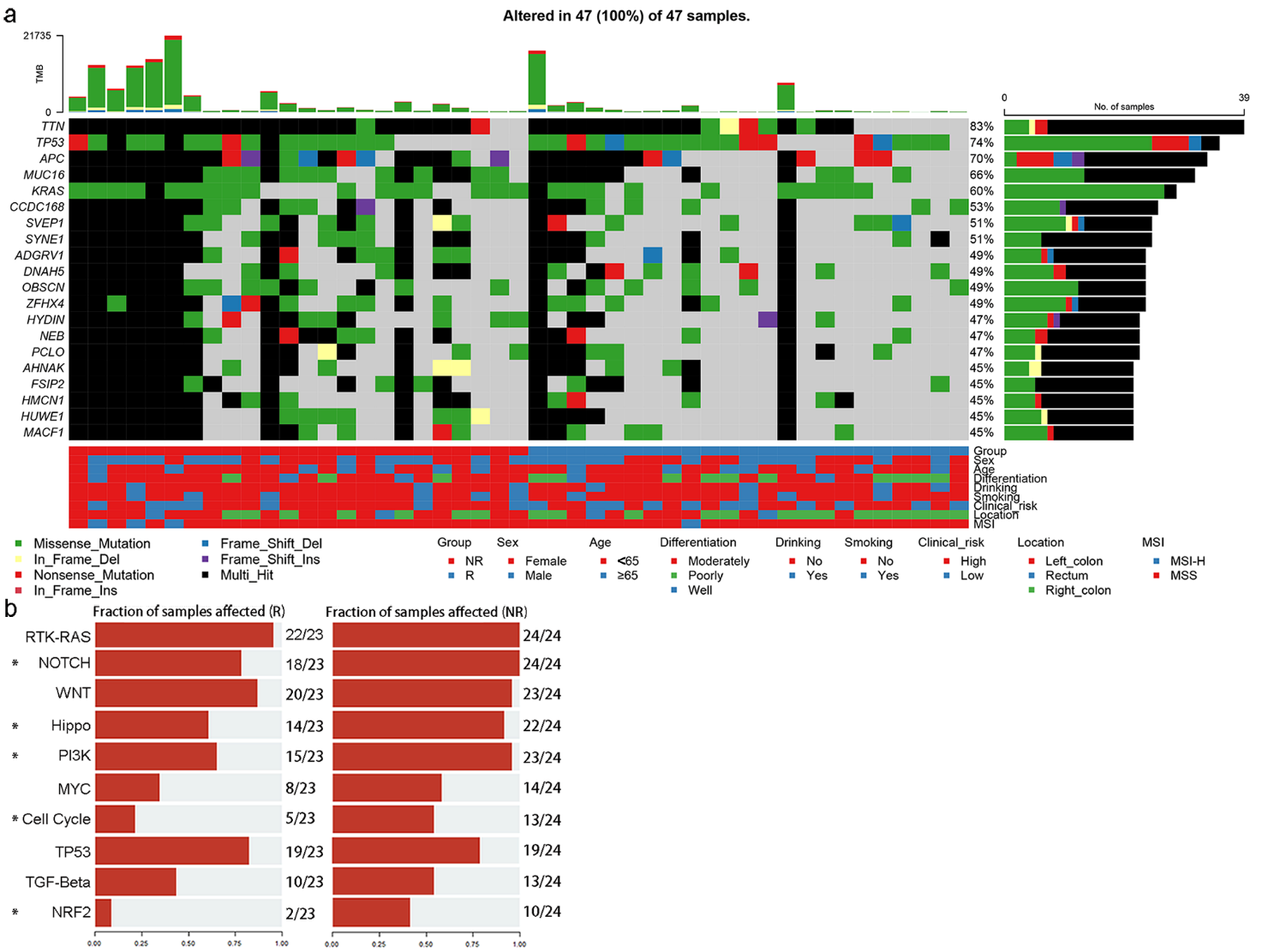


**Figure S2.** Genetic characteristics of CRC patients with or without recurrence.

a. Mutational landscape of all patients (top 20 genes). The samples were sorted by R and NR groups.

b. Comparison of the fraction of mutated samples in oncogenic signaling pathways between the R and NR groups (Fisher's exact test). *, P < 0.05. R, patients who relapsed within 2 years; NR, patients who did not relapse within 5 years.


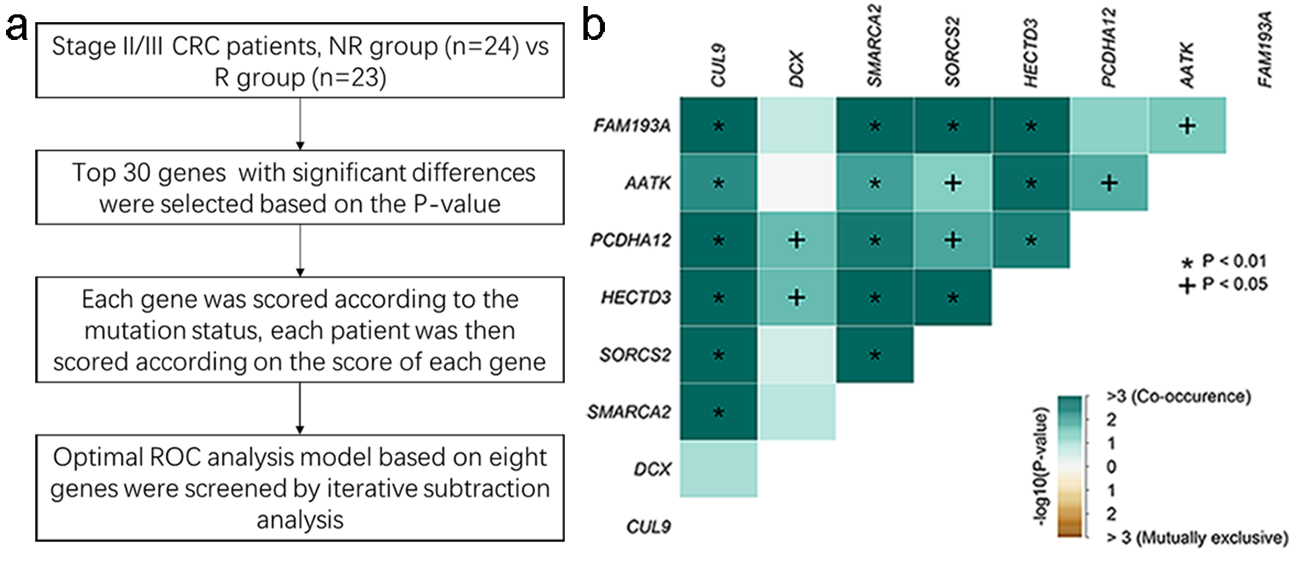


**Figure S3.** Construction of G8 score.

a. The flow chart showing the construction of G8 score. b. Interactions of the 8 genes. R, patients who relapsed within 2 years; NR, patients who did not relapse within 5 years.


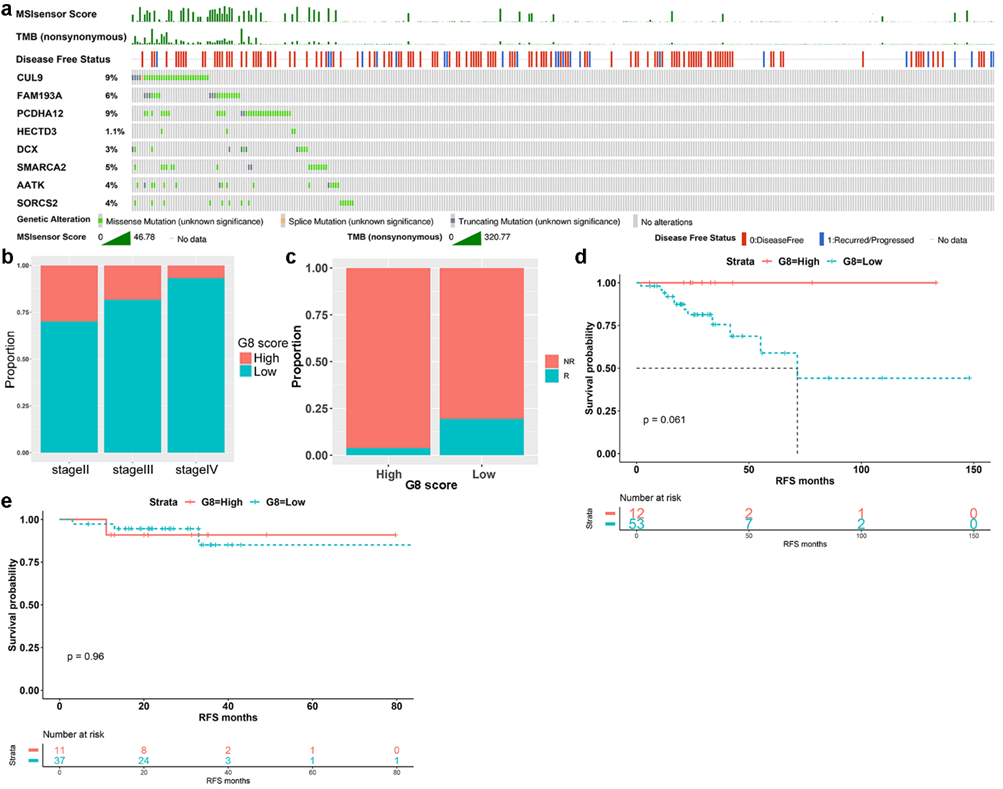


**Figure S4.** Validation of G8 score in the TCGA-COAD/READ cohort.

a. Distribution of the 8 genes in stage II/III CRC patients in the TCGA-COAD/READ cohort. b. Proportion of G8 high patients in the TCGA-COAD/READ cohort. c. Predictive recurrence performance of the G8 score in the TCGA-COAD/READ cohort. Kaplan–Meier curve of RFS for patients for CRC patients with stage IIB-III (d) and stage I (e) based on G8 score in the TCGA-COAD/READ cohort.


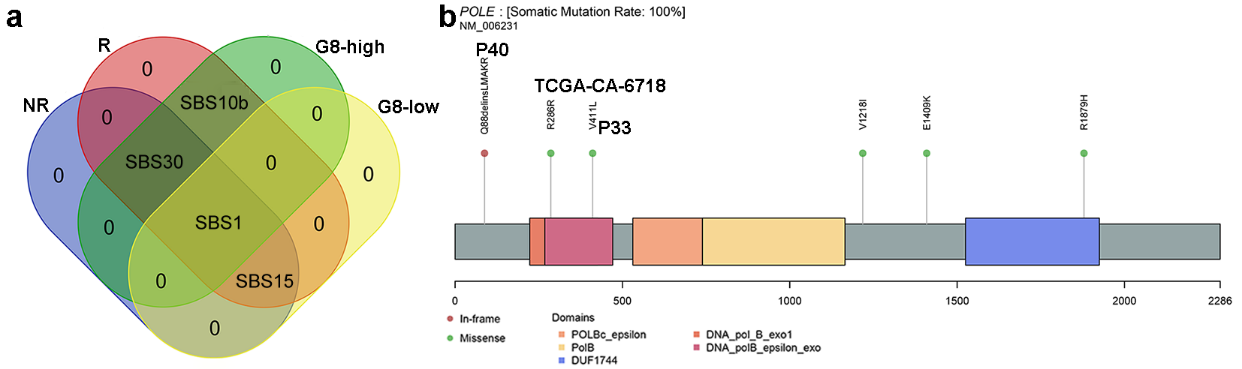


**Figure S5.** Mutational signature analysis of the samples with negative prediction errors.

a. Mutational signature that is unique or common in different groups. SBS10b: polymerase epsilon exonuclease domain mutations, SBS1: spontaneous or enzymatic deamination of 5-methylcytosine, SBS15: defective DNA mismatch repair, SBS30: deficiency in base excision repair due to inactivating mutations in NTHL1. b. Schematics of protein changes for *POLE* in the three samples with negative prediction errors.


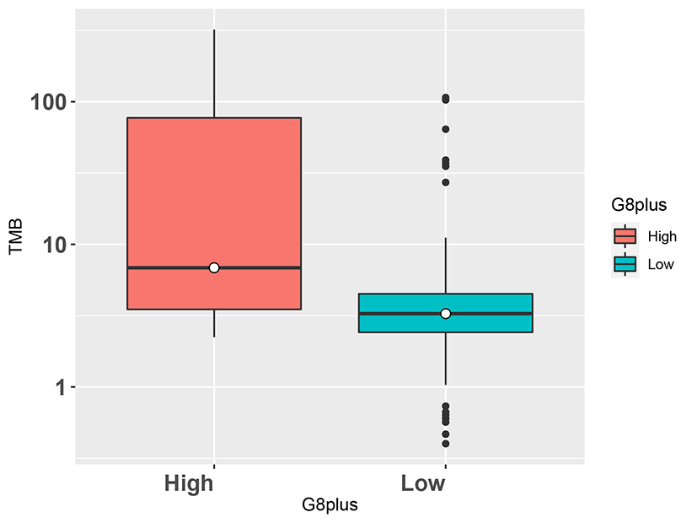


**Figure S6.** Comparison of TMB between G8plus high and G8plus low groups in the TCGA-COAD/READ cohort.


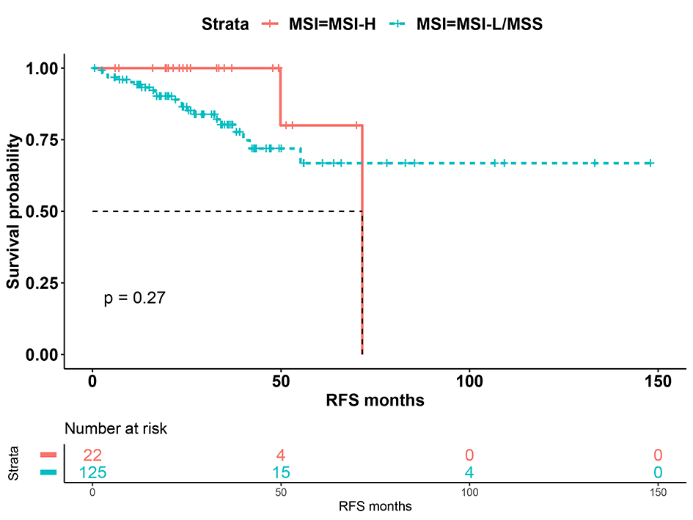


**Figure S7.** Kaplan–Meier curve of RFS for patients for stage II/III CRC patients based on MSI status in the TCGA-COAD/READ cohort.


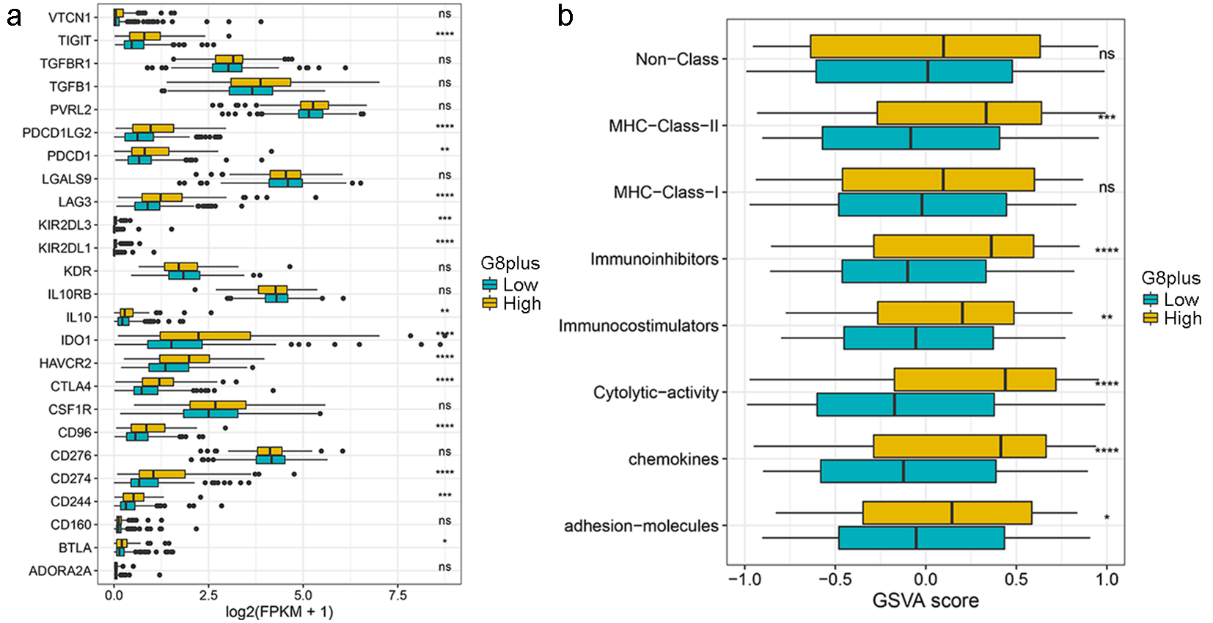


**Figure S8.** Expression of checkpoint genes (a) and immunological signatures (b) were compared between G8plus high and low groups. * p < 0.05; ** p < 0.01; *** p < 0.001.

**Table S1.** Predictive performance of the G8 score in our data.

|  | G8 low | G8 high | Total | Accuracy |
| --- | --- | --- | --- | --- |
| R | 21 | 2 | 23 | 95.74% |
| NR | 0 | 24 | 24 |  |

R, patients who relapsed within 2 years; NR, patients who did not relapse within 5 years.

**Table S2**. Interactions of the 8 genes in the TCGA-COAD/READ cohort.

| **A** | **B** | **Neither** | **A not B** | **B not A** | **Both** | **Log2 Odds Ratio** | **q-Value** |
| --- | --- | --- | --- | --- | --- | --- | --- |
| FAM193A | AATK | 327 | 14 | 10 | 6 | >3 | 0.002 |
| DCX | SORCS2 | 335 | 8 | 10 | 4 | >3 | 0.006 |
| CUL9 | FAM193A | 312 | 25 | 13 | 7 | 2.748 | 0.006 |
| FAM193A | PCDHA12 | 312 | 13 | 25 | 7 | 2.748 | 0.006 |
| CUL9 | AATK | 315 | 26 | 10 | 6 | 2.862 | 0.006 |
| PCDHA12 | AATK | 315 | 26 | 10 | 6 | 2.862 | 0.006 |
| CUL9 | SMARCA2 | 314 | 26 | 11 | 6 | 2.720 | 0.007 |
| PCDHA12 | SMARCA2 | 314 | 26 | 11 | 6 | 2.720 | 0.007 |
| PCDHA12 | SORCS2 | 316 | 27 | 9 | 5 | 2.701 | 0.014 |
| FAM193A | SORCS2 | 327 | 16 | 10 | 4 | >3 | 0.014 |
| PCDHA12 | DCX | 317 | 28 | 8 | 4 | 2.501 | 0.037 |
| CUL9 | PCDHA12 | 300 | 25 | 25 | 7 | 1.748 | 0.037 |
| AATK | SORCS2 | 330 | 13 | 11 | 3 | 2.791 | 0.043 |
| SMARCA2 | SORCS2 | 329 | 14 | 11 | 3 | 2.680 | 0.047 |
